# The crystallin alpha B (HSPB5)-tripartite motif containing 33 (TRIM33) axis mediates myocardial fibrosis induced by angiotensinogen II through transforming growth factor-β (TGF-β1)-Smad3/4 signaling

**DOI:** 10.1080/21655979.2022.2054913

**Published:** 2022-03-25

**Authors:** Tianwen Wei, Yingqiang Du, Tiankai Shan, Jiawen Chen, Dongwei Shi, Tongtong Yang, Jiankang Wang, Jun Zhang, Yafei Li

**Affiliations:** a Department of Cardiology, the Affiliated Suzhou Hospital of Nanjing Medical University, Suzhou Municipal Hospital, Gusu School, Nanjing Medical University, Suzhou, Jiangsu Province, China; b Department of Cardiology, the First Affiliated Hospital of Nanjing Medical University, Nanjing, Jiangsu Province, China; c Department of Intensive Care Unit, The Affiliated Changzhou NO. 2 People’s Hospital of Nanjing Medical University, Changzhou, Jiangsu Province, China

**Keywords:** Cardiac remodeling, myocardial fibrosis, TRIM33, HSPB5, TGF-β1, Smads

## Abstract

Myocardial fibrosis, a common pathological manifestation of cardiac remodeling (CR), often leads to heart failure (HF) and even death. The underlying molecular mechanism of the role of TRIM33 in Ang II–induced myocardial fibrosis is not fully understood. We found that TRIM33 was specifically upregulated in CFs and myocardial tissue after Ang II stimulation. Adult mice induced by Ang II were used as *in vivo* models, and Ang II–induced neonatal mouse primary cardiac fibroblasts (CFs) were used as *in vitro* models. The level of CF fibrosis *in vitro* was assessed by CF proliferation, migration, activation and extracellular matrix (ECM) synthesis. In addition, Masson staining, the heart weight/body weight (HW/BW) ratio and echocardiography were used to evaluate the *in vivo* effect of TRIM33. TRIM33 expression was specifically upregulated in CFs and myocardial tissue after Ang II stimulation. In *in vitro* experiments, we found that TRIM33 knockdown promoted Ang II–induced CF proliferation, while TRIM33 overexpression weakened Ang II–induced CF proliferation, migration, activation and collagen synthesis. Mechanistically, we showed that TRIM33, negatively regulated by HSPB5, mediated its antifibrotic effect by inhibiting the activation of TGF-β1 and its downstream genes, Smad3 and Smad4. Finally, TRIM33 overexpression suppressed fibrosis and promoted cardiac repair and functional recovery in Ang II–induced mice. Our results clearly establish that TRIM33 limits cardiac fibrosis by hindering CF proliferation, migration, activation and collagen synthesis. Enhancing these beneficial functions of TRIM33 by a targeting vector might be a novel therapeutic strategy for CR.

## Graphical abstract

**Figure:**
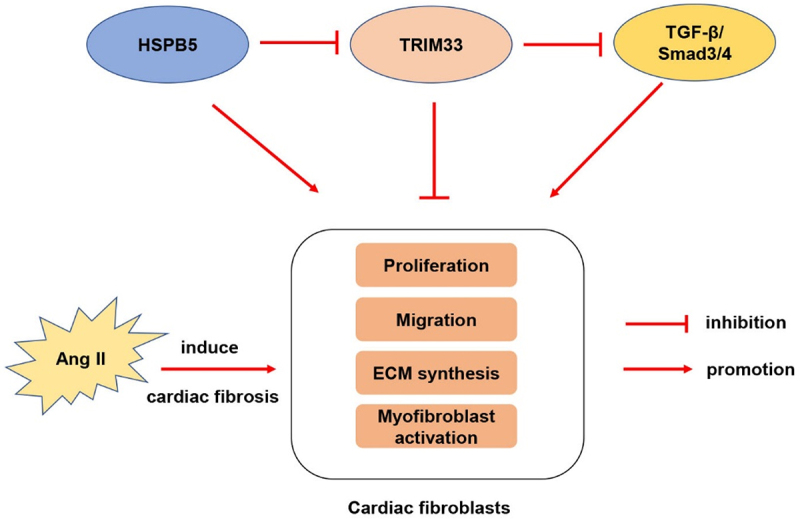

## Introduction

Heart failure (HF), an increasingly serious public health problem, has affected the health and life of more than 20 million people worldwide [1]. Despite major efforts in recent decades, the HF incidence rate and the mortality rate of HF patients have only slightly improved [2]. In recent years, the prevalence of HF has increased, and the 5-year mortality of symptomatic heart failure is even higher than that of various cancer patients [3]. Therefore, exploration of new therapies to improve the prognosis of patients with HF is urgently needed.

The most important feature of ventricular remodeling is CF-mediated myocardial fibrosis, which is the most important risk factor for the occurrence and development of HF [4]. In the early stage, myocardial fibrosis serves as a compensatory mechanism to maintain the structure and mechanical function of the damaged myocardium [5]. Nevertheless, persistent myocardial fibrosis can lead to excessive proliferation of CFs, aggravate the transformation of fibroblasts into myofibroblasts, and promote the synthesis of ECM [6]. During ventricular remodeling after HF, neurohumoral mechanisms such as renin-angiotensin-aldosterone system(RAAS), TGF-β1 and endothelin 1 enhance their response to mechanical and metabolic emergencies, resulting in increased activation and proliferation of cardiac fibroblasts [7]. Activated fibroblasts transform into α-smooth muscle actin (α-SMA) myofibroblasts, and myofibroblasts further promote collagen deposition, increase ventricular wall hardness and damage diastolic function [8]. Although modern medicine has made progress, few clinical treatments have been proven to be effective against cardiac fibrosis. Therefore, exploring the molecular mechanism of myocardial fibrosis after heart injury and finding alternative treatment strategies for cardiac fibrosis will provide a new direction for the prevention and treatment of CR.

The TRIM protein superfamily has been proven to be involved in a wide range of biological processes, including embryonic development, neurodegenerative diseases, cell cycle regulation and cancer [9,10]. As a member of the TRIM protein superfamily, TRIM33 has recently been proven to be an E3 ubiquitin ligase that can mediate the ubiquitination of Smad4 and induce its export from the nucleus to inhibit the transcriptional activity of Smads [11]. In addition, TRIM33 has been shown to act as a negative conditioner of TGF-β1 in different cell types [12]. TGF-β1 is a major fibrogenic gene that can modify Smads by phosphorylation and promote their translocation to the nucleus. This gene has been proven to mediate the occurrence and progression of almost all fibrotic diseases [13]. HSPB5, as an important member of the small heat shock proteins (sHSPs) family, is widely expressed in different tissues and plays an important role in biology such as protein degradation, cytoskeleton stability and apoptosis [14]. Previous study has shown that HSPB5 can reduce the expression of Trim33 and affect the progression of pulmonary fibrosis [12]. However, the regulatory role of TRIM33 and HSPB5 in myocardial fibrosis is still unclear.

In the study presented here, we successfully constructed Ang II–induced fibrosis models *in vivo* and *in vitro* and showed that TRIM33 was dysregulated in Ang II–induced ventricular remodeling models. We hypothesized that TRIM33 is implicated in the progression of cardiac fibrosis by regulating the activation and proliferation of CFs. Based on the cardiac fibrosis model, we detected that TRIM33 overexpression could markedly attenuate the extent of cardiac fibrosis in Ang II–induced *in vivo* and *in vitro* models. Mechanistically, we demonstrated that TRIM33 overexpression could suppress cardiac fibrosis by inhibiting CF activation, proliferation and ECM production via TGF-β1/Smad3/4 signaling. Moreover, these biological functions of TRIM33 in blocking myocardial fibrosis could be reversed by HSBP5. Accordingly, we aim to detect the effect of TRIM33 on cardiac fibrosis and the possible mechanisms for regulating CF proliferation, activation and ECM accumulation, thereby improving outcomes in individuals suffering from HF.

## Methods

### The human protein atlas analysis

Human protein atlas (HPA) database (https://www.proteinatlas.org/) is a public portal, belonging to the human proteomics research project, which maps the protein expression and localization of a large number of normal and cancer tissues and organs. It aims to provide tissue and cell distribution information of all 24,000 human proteins. Using this database, we analyzed the expression of TRIM33 in different human organs and tissues, and further analyzed its expression in different cells of heart tissue.

### Animal model

The *in vivo* study was approved by the medical ethics committee of Nanjing Medical University. Eight-week-old and 1-3-day-old C57BL/6 mice were purchased from the Animal Core Facility of Nanjing Medical University (Nanjing, China) and raised in a specific pathogen-free environment. Eight-week-old adult male mice were continuously injected subcutaneously with Ang II (1.5 mg/kg/day) for 28 days to establish an *in vivo* model of cardiac fibrosis as previously described [15], and the mice in the control group were injected subcutaneously with the same amount of normal saline. Cardiac function was evaluated by transthoracic echocardiography after continuous injection for 28 days, and Masson staining was conducted to evaluate the level of cardiac fibrosis. qRT-PCR was also conducted to detect the levels of TRIM33 and CR indicators (ANP, MYH7 and Col 1).

### Recombinant adenovirus and plasmid

Recombinant AAV9-TRIM33 (adeno-associated virus carrying mRNA for mouse TRIM33) and AAV9-Control (expressing enhanced green fluorescent protein) were purchased from Genebay Company (Nanjing, China). The recombinant AAV9-TRIM33 and AAV9-control were injected through the tail vein at 2 × 10^12^ v.g./mouse one week before the first treatment with Ang II. The TRIM33 and HSPB5 overexpression plasmids were also purchased from Genebay Company (Nanjing, China) (source of genes: TRIM33: NM_053170.3:587–4009 Mus musculus tripartite motif-containing 33 (Trim33), transcript variant 1, mRNA; HSPB5: NM_009964.3:307–834 Mus musculus crystallin, alpha B (Cryab), transcript variant 2, mRNA). The plasmids for mouse TRIM33 shRNA (TRIM33 shRNA) and vector (expressing enhanced green fluorescent protein) were purchased from Genebay Company (Nanjing, China), and the target sequence of shRNA for mouse TRIM33 was 5’- GGACGAAGATGATGGTGAA −3’.

### Isolation of CFs, culture, and transfection

A total of 50 neonatal (1–3 days old) C57BL/6 suckling mice were euthanized and disinfected with 75% ethanol, and cardiac fibroblasts were isolated. Briefly, the ventricular myocardial tissue of the 50 neonatal mice was collected and shredded. Then, the preprepared myocardial digestion solution containing 0.6% trypsin (Sigma, USA) and 0.4% collagenase (Worthington, USA) was added for digestion. After 6–8 minutes each time, the digestible liquid was removed, and 4 ml of horse serum (Biological Industries, Israel) was added to terminate the digestion. After the myocardial tissue was completely digested, the mixture was centrifuged and incubated in a culture containing 10% fetal bovine serum for 40 min. Then, the supernatant was aspirated, and CFs were harvested by differential adhesion methods and cultured in CF culture medium consisting of Dulbecco’s modified Eagle’s medium (DMEM, Gibco, USA) with 10% fetal bovine serum and 1% penicillin-streptomycin-neomycin. Two to three generations of CFs were used in all studies.

The CFs were treated with Ang II (1 mmol/L) for 48 h (Sigma, St. Louis, MO, USA) to establish the cellular model of cardiac fibrosis in the presence or absence of TRIM33/HSPB5 overexpression plasmids and TRIM33 shRNA. RNA transfection was performed according to the manufacturer’s instructions by using Lipofectamine 2000 Transfection Reagent (Invitrogen) [16].

### Quantitative real-time polymerase chain reaction (qRT-PCR) analysis

Myocardial tissue harvested from HF animal model or CFs treated with Ang II were added to 1 ml of TRIzol, ground and transferred into a 1.5 ml EP tube. After measuring the concentration, the extracted total RNA(20 μg) was further reverse transcribed into cDNA. The reverse transcription reaction conditions were as follows: 37°C for 15 minutes, 85°C for 5 seconds, and storage at 4°C until use. Quantitative real-time PCR was performed according to the instructions of the kit to determine the expression of the target gene. The reaction conditions were as follows: 95°C for 5 min, followed by 40 cycles of 95°C for 10s and 60°C for 30s, with a final dissociation stage. The specific primer sequences are shown in Table 1.

**Table 1.** qRT-PCR primer sequences

Genes	Genebank Accession	Sequences	
TRIM33	NM_001079830	Forward	GCCCGATGTGATCCGT
		Reverse	GGAGGAACTTGCCCAACTA
ANP	NM_008725	Forward	TGACGGACAAAAGCTGAGA
		Reverse	CAGGGTGATGGAGAAGGAG
MYH7	NM_080728	Forward	ATCCCAGCTCCAGACAGAA
		Reverse	TCAGCTCCTCAGCCATCA
Col I	NM_007742	Forward	TGTTCACCATCATCCCAAG
		Reverse	ACAATTTCGACCTGGCAA
Col III	NM_009930	Forward	CCGGGTGGTATGGAAGT
		Reverse	GGTTGAAGATGAGCAGGTG
HSPB5	NM_009964	Forward	TCCAGCAGGTTATCCACAG
		Reverse	GGTGAGGTCAGGGGTTTT
GAPDH	NM_008084	Forward	TGTTTCCTCGTCCCGTAGA
		Reverse	ATCTCCACTTTGCCACTGC

### Western blot analysis

The tissues or CFs of different treatment groups were lysed on ice using the preprepared lysis buffer (1 ml containing 1 µl of protease inhibitor, 5 µl of PMSF and 10 µl of phosphatase inhibitor). The protein concentration was detected by the BCA protein assay kit (Thermo Fisher Scientific, USA). Then, the total protein of tissues or cells was separated by SDS-PAGE (EpiZyme Scientific, China). The primary antibodies applied in the study included TRIM33 (CST, 90051S, diluted 1:1000), HSPB5 (CST, 45844S, diluted 1:1000), tubulin (CST, 2148S, diluted 1:1000), TGF-β1 (CST, 84912S, diluted 1:1000), α-SMA (CST, 19245S, diluted 1:1000), β-catenin (CST, 8480S, diluted 1:1000), FOXO3a (CST, 12829S, diluted 1:1000), Col 1 (CST, 72026S, diluted 1:1000), p-Smad3 (CST, 9520S, diluted 1:1000), Smad3 (CST, 9523S, diluted 1:1000), p-Smad4 (Invitrogen, PA5-12,695, diluted 1:1000), Smad4 (CST, 46535S, diluted 1:1000), and the internal reference GAPDH (CST, 5174S, diluted 1:1000). After incubation with primary antibodies overnight, the membranes were incubated with the secondary goat anti-rabbit or goat anti-mouse HRP conjugated antibody (Thermo Fisher Scientific, diluted 1:5000) for 2 hours. The membranes were then washed 3 times with TBST for 20 minutes each time. Finally, the membranes were detected and statistically analyzed.

### Cell migration assay

CFs at a density of 3 × 10^5^ were uniformly distributed in each well of the 24-well plate, and induced by Ang II. When the cells grew to 50% confluence, CFs cells were respectively transfected with TRIM33 knockdown plasmid and overexpression plasmid. 24 hours after transfection, vertical scratches were made with a 20 ml sterile gun head, and PBS was used to remove the scratched cell fragments. Scratch regions were recorded by microscopy. Cell culture was continued 24 hours after obtaining microscope photos and quantifying the migration area.

### Cell proliferation assay

According to the operating instructions of the EdU kit (Thermo Fisher Scientific, USA), 12 hours before CF staining, a solution with a concentration of 4 µl/ml was added to the cell culture medium. The cell culture medium was discarded, the cells were washed with precooled PBS 3 times, paraformaldehyde was added, the cells were fixed for 20 minutes, and then, 0.2% Triton solution was added to disrupt the membrane. After goat serum was added for blocking for one hour, a-SMA primary antibody (CST, 19245S, diluted 1:300) was incubated at 4°C and slowly shaken overnight. The next day, the primary antibody was discarded, and the secondary antibody was incubated at room temperature for 2 hours after 3 washes with PBS. Next, the staining solution was prepared according to the EdU instructions, added to the cell culture plate and shaken slowly at room temperature for 45 minutes. After absorbing the EdU staining solution, the cells were washed with PBS 3 times, and the nuclear staining agent DAPI (Sigma-Aldrich, F6057, diluted 1:300) was added and shaken slowly at room temperature for 5 minutes. Finally, the proliferation levels of CFs in different treatment groups were photographed and the randomly selected visual fields were counted by Zeiss microscope (Carl Zeiss Microscopy, Jena, Germany) [17].

### Echocardiography analyses

Mice anesthetized with isoflurane were examined by echocardiography (Visuasonics, Ontario, California). The parameters of left ventricular inner diameter (lvid), left ventricular shortening fraction (FS) and left ventricular ejection fraction (EF) were measured by M-mode images of parasternal short axis horizontal papillary muscle intervention. The weight of the mice was recorded before euthanasia, and then, the heart was dissected and weighed to calculate the ratio of heart weight to body weight (HW/BW) [18].

### Masson’s trichrome staining

A Masson staining kit was used to dye the 5-μm tissue sections according to the following steps: Weigert iron hematoxylin staining solution was added and incubated for 10 min, and distilled water was added and incubated for 10 min. The cells were incubated with blue returning solution for 3 min and washed with distilled water for 1 min. Then, the cells were dyed with Ponceau fuchsin staining solution for 5 min and washed with a weakly acidic working solution for 1 min. The phosphomolybdic acid solution was used for differentiation for 2 min and weakly acidic solution was incubated for 1 min. The cells were dyed with aniline blue staining solution for 2 min and then washed with a weakly acidic working solution for 1 min. The sections were rapidly dehydrated with 95% ethanol 3 times, anhydrous ethanol 3 times, cleared with xylene for 2 minutes each time, and sealed with resin. A small amount of neutral resin was dipped onto the tissue surface on the slide, the cover glass was covered from right to left, the cover glass was avoided, the bubbles between the slides were discharged, and the slide was dried in a 37°C oven for 1 ~ 2 hours. The images were then taken under a stereomicroscope and a forward fluorescence microscope. The area of cardiac fibrosis was quantified by calculating the fibrotic area versus the total ventricular area using ImageJ software. The average fibrotic area was calculated from 5 slides in each animal.

### Statistical analysis

The experimental data were analyzed by SPSS 21 statistical software. The values were expressed as ‘mean ± SEM’ using graphpad prism 7.0 software. All experiments were performed with three sets of biological replicates at least. The comparisons between two groups were performed by student’s t test. More than 2 groups were analyzed by one-way ANOVA with the post hoc Tukey test. P < 0.05 was considered statistically significant.

## Results

In this study, we hypothesized that TRIM33 participates in mediating myocardial fibrosis induced by Ang II. We proved that TRIM33, which is mediated by HSPB5, could protect against myocardial fibrosis through TGF-β1-Smad3/4 signaling.

### TRIM33 expression is upregulated in Ang II-induced myocardial tissue and CFs

**Figure 1.**
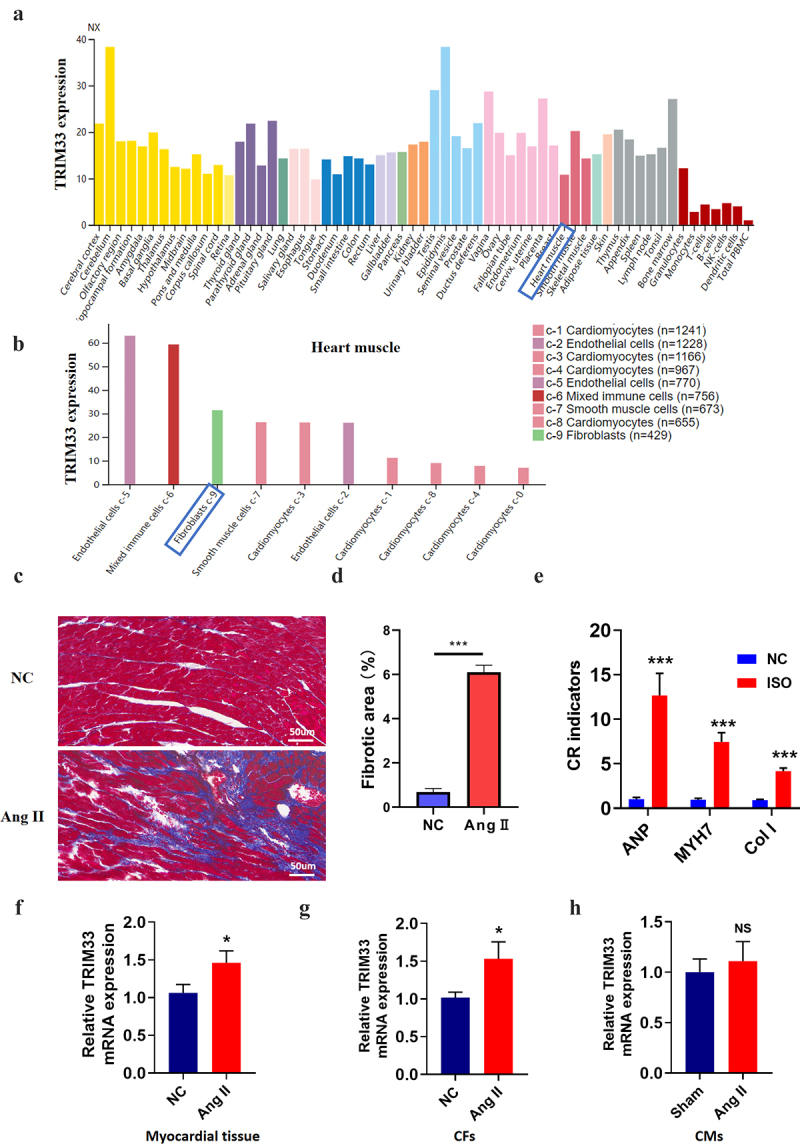
TRIM33 was upregulated in Ang II–induced *in vivo* and *in vitro* models. a-b. The proteinatlas (https://www.proteinatlas.org/) analysis of TRIM33 expression characteristics in human organs and cells in herat muscule. NX: the consensus normalized expression; c-d. Masson staining was performed to determine the scar size between Ang II induced mice and control mice to verify the cardiac remodeling model (n = 6 in each group). e.The qRT-PCR analysis was performed to determine the expression of fibrosis marker genes ANP, MYH7 and Col 1 (n = 6 per group). f-h. The qRT-PCR was used to examine the TRIM33 expression in Ang II induced myocardial tissue, CFs and CMs respectively. Each experiment was conducted three times independently (n = 6 per group). *P < 0.05, ***P < 0.001, ns: No statistical significance.

Through protein atlas analysis in humans, we found that TRIM33 was expressed in various organs of the human body, including myocardial tissue (Figure 1a). In addition, TRIM33 was enriched in endothelial cells, immune cells, CFs and cardiomyocytes, indicating that TRIM33 is not a tissue-specific gene (Figure 1b). To investigate the role of TRIM33 in cardiac fibrosis, we first generated a mouse CR model using Ang II. After 28 days of Ang II administration, Masson’s trichrome staining showed that the area of cardiac fibrosis in the Ang II-treated mice was significantly higher than that in the NC mice (Figure 1c,d). In addition, the expression of the fibrosis marker genes ANP, MYH7 and Col 1 was obviously induced in the Ang II group mice, indicating that the CR model was successfully constructed (Figure 1e). Subsequently, RT-PCR analysis revealed that TRIM33 expression was significantly upregulated in the Ang II-treated myocardial tissue and CFs (figure 1f,g), while TRIM33 expression was not changed in the Ang II-treated CMs (Figure 1h).

### TRIM33 impedes Ang II-induced CF proliferation, migration, activation and collagen synthesis

**Figure 2.**
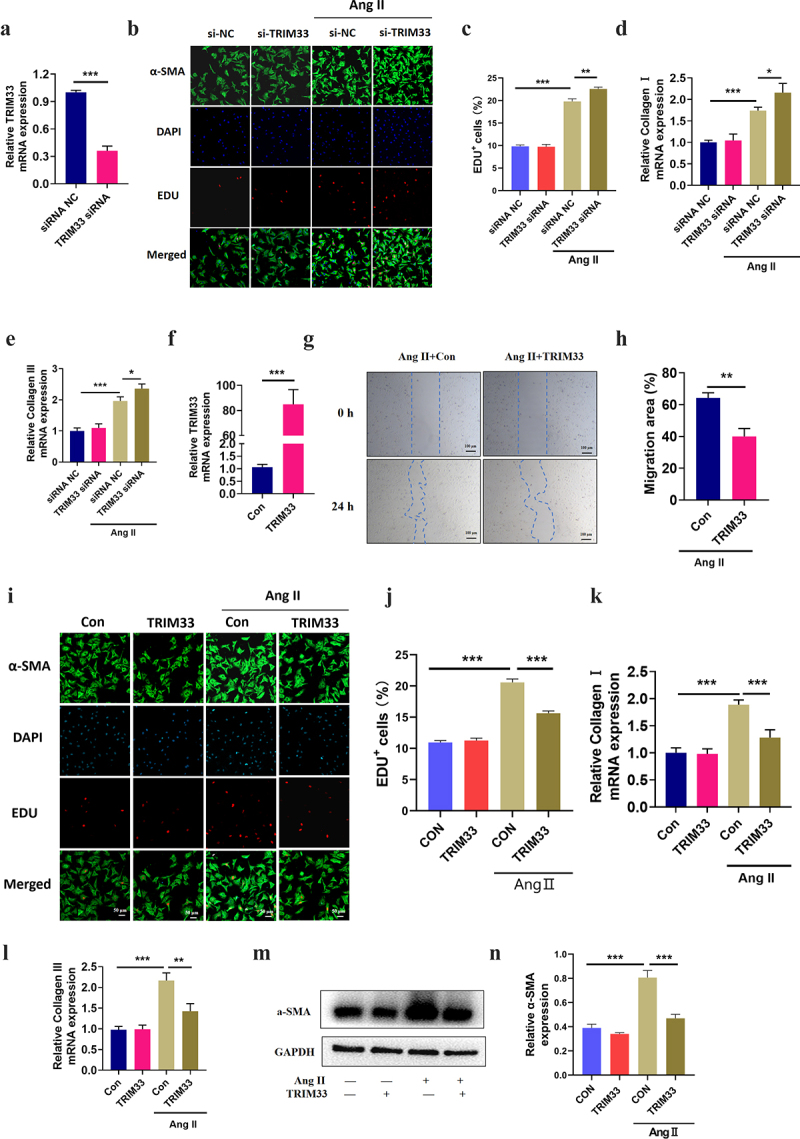
TRIM33 blocks Ang II induced CF fibrosis *in vitro*. a. The qRT-PCR analysis was performed to confirm the knockdown efficiency of TRIM33 shRNA. b-c. EDU immunofluorescence staining was conducted to detect the proliferation rate of CFs. d-e. The qRT-PCR analysis was used to clarify the expression of ECM genes Col 1 and Col 3 in different groups. f. The qRT-PCR analysis was performed to confirm the overexpression efficiency of TRIM33 overexpression plasmid. g-h. The migration assay was used to confirm the migration of CFs after TRIM33 overexpression. i-j. EDU immunofluorescence staining was conducted to detect the proliferation rate of CFs in four groups. k-l. The qRT-PCR analysis was used to clarify the expression of ECM genes Col 1 and Col 3 in different groups. m-o. Western blot analysis was performed to detect the a-SMA expression in response to Ang II stimulation. Each experiment was conducted three times independently. *P < 0.05, **P < 0.01, ***P < 0.001, ns: No statistical significance.

As TRIM33 expression is upregulated in fibrotic myocardial tissue and CFs induced by Ang II *in vitro* and *in vivo*, we next explored the role of TRIM33 in fibrosis in activated CFs. We first constructed a TRIM33 knockdown plasmid (TRIM33 shRNA) and transfected it into CFs for 48 hours, and RT-PCR analysis was used to confirm the knockdown efficiency of TRIM33 shRNA (Figure 2a). By EdU immunofluorescence staining, we found that Ang II promoted the proliferation of CFs, while knocking down TRIM33 further enhanced the proliferation of CFs (Figure 2b,c). Similarly, TRIM33 inhibition additionally elevated the expression of the ECM genes Col 1 and Col 3 in the Ang II-treated CFs (Figure 2d,e). The above results suggest that TRIM33 may be a protective gene in the process of cardiac fibrosis.

To determine whether TRIM33 inhibits Ang II–induced fibrosis in CFs, we constructed a TRIM33 overexpression plasmid and verified its transfection efficiency in neonatal mouse CFs (figure 2f). Cell migration assay showed that TRIM33 overexpression significantly inhibited the migration of the Ang II–induced CFs (Figure 2g,h). Next, EdU immunofluorescence staining revealed that the proliferation level of the Ang II–induced CFs was significantly blocked by TRIM33 overexpression, and a-SMA was activated in Ang II–induced CFs, which showed that the fluorescence brightness of green contour was significantly higher than that of the control group, while TRIM33 could inhibit Ang II–induced a-SMA activation (Figure 2i,j). Furthermore, overexpression of TRIM33 in CFs decreased the expression of the ECM genes Col 1 and Col 3 (Figure 2k,l) and reduced the protein expression of the CF activation marker gene a-SMA in response to Ang II stimulation (Figure 2m-o).

### TRIM33 mediated by HSPB5 suppresses fibrogenic TGF-β1-Smad3/4 signaling in CFs

**Figure 3.**
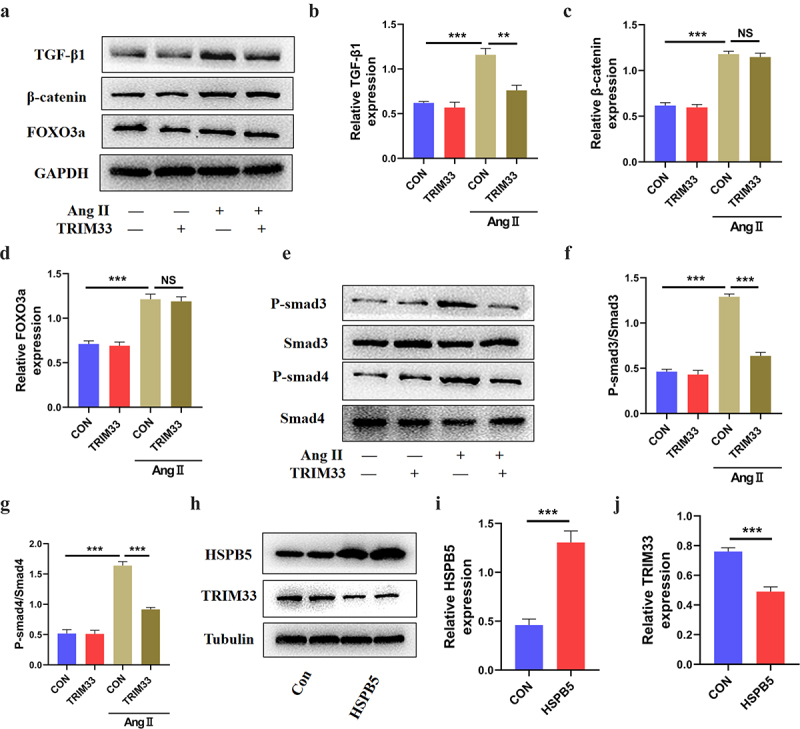
HSPB5-TRIM33 axis suppresses TGF-β1-Smad3/4 signaling in CFs. a-d. Western blot analysis was used to detect the expression of TGF-β1, β-catenin and FOXO3a in CFs after TRIM33 overexpression. e-g. The P-smad3/Smad3 and P-smad4/Smad4 level after TRIM33 overexpression was determined by Western blot. h-j. Western blot analysis was used to detect the expression of HSPB5 and TRIM33 in HSPB5 overexpressed CFs. Each experiment was conducted three times independently. **P < 0.01, ***P < 0.001, ns: No statistical significance.

TGF-β1-Smad3/4 signaling has an important role in the process of fibroblast fibrosis and is considered to be a core pathway for the progression of CR [19,20]. Recently, the fibrotic regulatory genes TGF-β1, β-catenin and FOXO3a have been shown to be regulated by TRIM33 in other diseases [12,21,22]. To clarify whether TRIM33 can regulate TGF-β1, β-catenin and FOXO3a signaling in CFs, we cultured neonatal mouse CFs with Ang II with or without the TRIM33 overexpression vector. Western blot analysis results showed that TRIM33 could significantly inhibit TGF-β1 expression but had no effect on β-catenin and FOXO3a expression induced by Ang II (Figure 3a-d). Both Smad3 and Smad4 are downstream genes of TGF-β1 and play an important role in the process by which TGF-β1 promotes fibrosis. As shown in Figure 3e-g, the elevation of P-Smad3/Smad3 and P-Smad4/Smad4 levels induced by Ang II was significantly inhibited by TRIM33 overexpression in CFs. Moreover, TRIM33 and HSPB5 have been proven to be important regulatory genes of TGF-β1 [12]. In addition, TRIM33 was shown to be negatively regulated by HSPB5 in non-small-cell lung cancer cells (A549 cells) [12]. Here, we also demonstrated that overexpression of HSPB5 reduced TRIM33 expression in CFs (Figure 3h-j).

### TRIM33 eliminates the further deterioration of fibrosis induced by HSPB5 in Ang II-treated CFs

**Figure 4.**
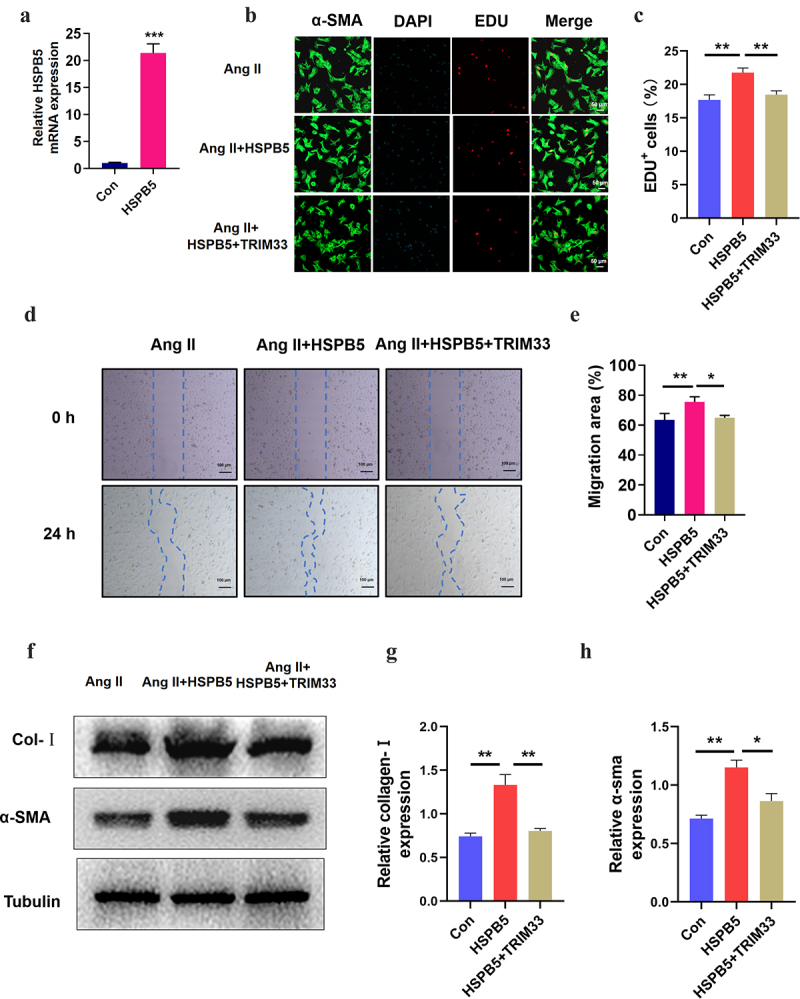
TRIM33 attenuates the fibrosis induced by HSPB5. a. The qRT-PCR analysis was used to clarify the expression of HSPB5. b-c. EDU immunofluorescence staining was conducted to detect the proliferation rate of CFs. d-e. The migration assay was used to confirm the migration of CFs in four groups. f-g. The Coll I and a-SMA expression level was determined by Western blot. Each experiment was conducted three times independently. *P < 0.05,**P < 0.01, ***P < 0.001.

Next, to further verify the role of the HSPB5-TRIM33 axis in Ang II–induced fibroblasts, we treated CFs with the HSPB5 overexpression plasmid with or without transfection of the TRIM33 overexpression plasmid for 48 hours. The transfection efficiency of the HSPB5 overexpression plasmid was verified by RT-PCR analysis (Figure 4a). EdU immunofluorescence experiments indicated that HSPB5-induced CF proliferation could be significantly reversed by TRIM33 overexpression in the Ang II-treated CFs (Figure 4b,c). Similarly, HSPB5 further promoted Ang II–induced CF migration, but TRIM33 overexpression blunted the CF migration increased by HSPB5 overexpression (Figure 4d,e). Finally, HSPB5 increased the protein expression of collagenase-1 and a-SMA, while TRIM33 overexpression impaired this effect of HSPB5 (figure 4f,g).

### *TRIM33 protects against Ang II-induced cardiac fibrosis and cardiac function* in vivo

**Figure 5.**
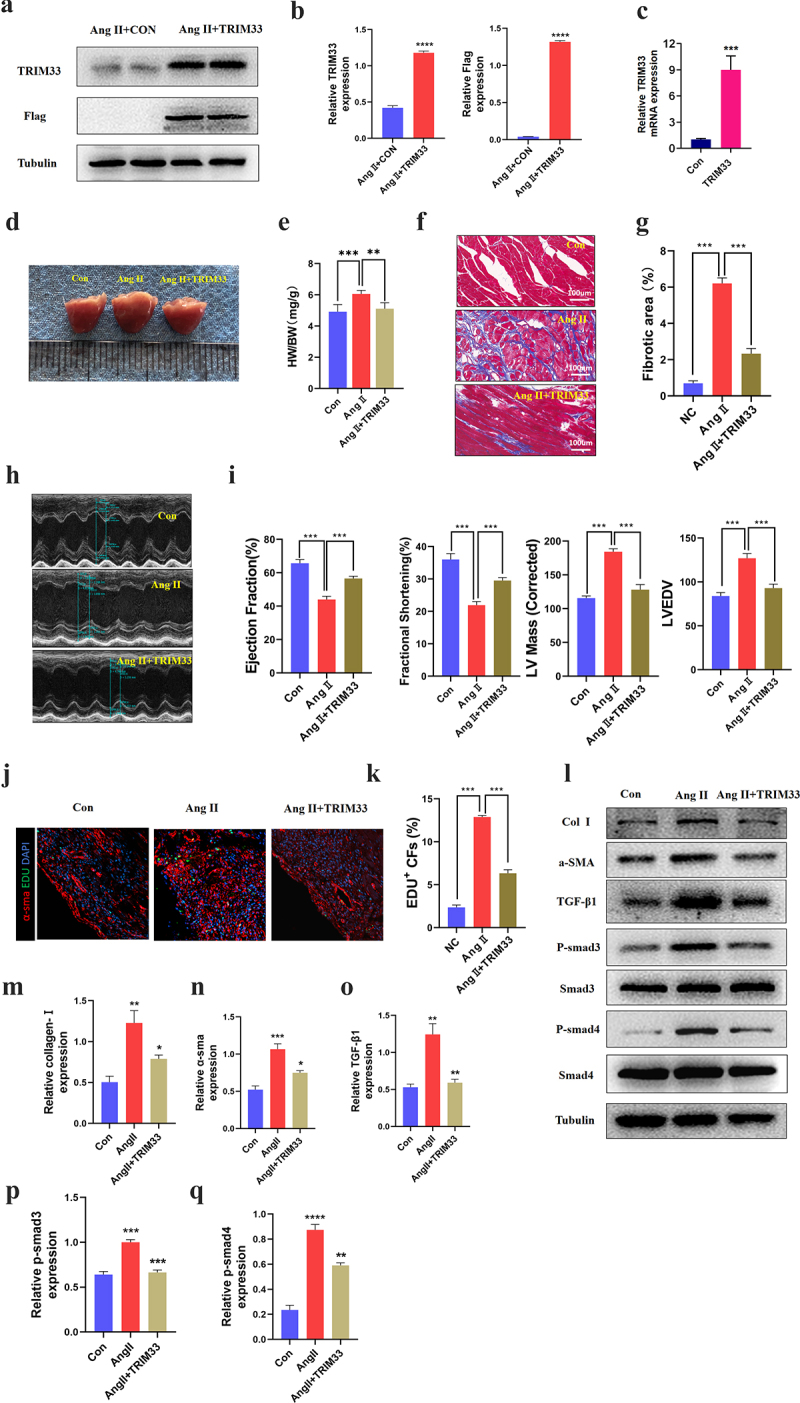
TRIM33 protects against Ang II–induced cardiac injury *in vivo*. a-b. Western blot analysis was used to detect the expression of TRIM33 and Flag. c. The qRT-PCR analysis was used to clarify the expression of TRIM33. d-e. The heart weight/body weight (HW/BW) ratio was determined after TRIM33 overexpression (n = 6 per group). f-g. Masson staining was used to confirm the fibrosis scar area after TRIM33 overexpression (n = 6 per group). h-i. Echocardiographic analysis was conducted to exam the cardiac function (n = 6 per group). j-k. EDU immunofluorescence staining was used to count the proportion of EDU^+^ CFs in Con and TRIM33 overexpression mice (n = 6 per group). l-q. The Coll I, a-SMA and TGFβ1-Smad3/4 signaling expression level was determined by Western blot. Each experiment was conducted three times independently. *P < 0.05,**P < 0.01, ***P < 0.001.

To explore the contribution of TRIM33 to cardiac fibrosis and cardiac function *in vivo*, we injected the TRIM33 overexpression adeno-associated virus 9 (AAV9-TRIM33)-treated mice and the sham-treated mice with Ang II (15 mg/kg/day subcutaneously for 28 days) [15]. Western blot and RT-PCR analysis was used to confirm the successful transfection of AAV9-TRIM33 in myocardial tissue 14 days after transfection (Figure 5a-c). As shown in Figure 5d,e, Ang II treatment markedly increased the heart weight/body weight (HW/BW) ratio, while it was decreased in the Ang II+AAV9-TRIM33-treated mice. Masson staining results showed that Ang II treatment induced extensive fibrosis, whereas slight fibrotic changes were observed in the Ang II+AAV9-TRIM33-treated mice (figure 5f,g). In addition, echocardiographic analysis demonstrated obviously reduced Left Ventricular Ejection Fraction (LVEF) and fractional shortening (FS) in the Ang II-treated mice, whereas they were preserved in the Ang II+AAV9-TRIM33-treated mice (Figure 5h,i). Moreover, EdU immunofluorescence staining indicated that the proportion of EDU^+^ CFs in the Ang II group was significantly higher than that in the control group but was significantly blocked by TRIM33 overexpression (Figure 5j,k). Consistent with the above observations, TRIM33 overexpression also abolished the expression of the ECM synthesis-related protein Col 1 and the CF activation marker a-SMA induced by Ang II in cardiac tissue. Further, the expression of TGFβ1-Smad3/4 signaling was also downregulated with TRIM33 overexpression *in vivo* (Figure 5l,q). Thus, in addition to eliminating Ang II–induced CF proliferation and ECM synthesis, TRIM33 overexpression protects against Ang II–induced heart size, cardiac dysfunction and myocardial fibrosis *in vivo*.

## Discussion

Cardiac fibrosis caused by various cardiac dysfuntion is characterized by CF proliferation, activation and the production of excessive ECM in the cardiac interstitium [23,24]. In the progression of all types of myocardial injury, such as hypertension, myocardial infarction or cardiotoxic drugs, neurohumoral factors are activated and trigger CFs to differentiate into myofibroblasts. These factors aggravate fibrotic tissue formation and eventually lead to increased ventricular stiffness, diastolic dysfunction and even HF [25]. In this study, we found that the expression of TRIM33 was upregulated in Ang II–induced fibrotic myocardium and CFs, but there was no significant change in cardiomyocytes, indicating that Trim33 may be a functional therapeutic target in the occurrence and development of myocardial fibrosis.

TRIM33 is involved in a broad range of cellular processes, including cell cycle arrest, oxidative stress, apoptosis, ubiquitination, and fibrosis [12^,26–28^]. For instance, overexpression of TRIM33 could suppress the proliferation, migration and invasion of clear cell renal cell carcinoma cells [26]. TRIM33 attenuated oxidative stress-induced osteoblast apoptosis in osteoporosis by blocking FOXO3a ubiquitination and degradation [22]. Recently, Pierre-Marie Boutanquoi et al. revealed that the production of TRIM33 is elevated during the progression of pulmonary fibrosis, and inhibition of TRIM33 in the lung aggravated BLM-induced fibrosis in mice. Similar to this finding, our results also demonstrated that TRIM33 overexpression protects against Ang II–induced cardiac fibrosis and cardiac function *in vivo* and *in vitro*, suggesting that TRIM33 plays a protective role in the progression of cardiac fibrosis.

In terms of mechanistic exploration, studies have shown that TRIM33 regulates the core genes related to fibrosis, including FOXO3, β-catenin, TGF-β1 and Smads [12,21,22]. We explored the known downstream genes of TRIM33 in activated CFs. The results showed that only TGF-β1 expression decreased after overexpression of TRIM33, rather than β-catenin and FOXO3, suggesting that TRIM33 plays antifibrotic roles independent of β-catenin and FOXO3. TGF-β1 signaling is an important regulatory pathway in the progression of cardiac fibrosis, and TGF-β1 expression is significantly activated and can promote fibrosis through the downstream Smad pathway post-cardiac injury.

Furthermore, a recent study showed that inhibition of TRIM33 in the lung aggravates BLM-induced fibrosis in mice by negatively regulating TGF-β1 downstream signaling [12]. Similar to these findings, our results also demonstrated that TRIM33 overexpression protects against Ang II–induced cardiac fibrosis and cardiac function *in vivo* and *in vitro*, suggesting that TRIM33 plays a protective role in the progression of cardiac fibrosis. In a preclinical fibrosis model, targeted knockdown of TGF-β1 has achieved certain curative effects and progress. However, direct knockout of TGF-β1 signals may lead to impaired physiological function and adverse reactions, including liver and cardiotoxicity [29]. In this study, although the expression of TRIM33 in the Ang II–induced group increased moderately compared with that in the control group, we believe that this effect is only a self-protective feedback increase and is not sufficient to play an antifibrotic role. When the expression of TRIM33 increases exponentially in CFs, it may have a significant antifibrotic role. Thereover, In the *in vivo* and *in vitro* models, we proved that overexpression of TRIM 33 significantly inhibited the expression of TGF-β1 induced by Ang II in CFs, thereby disrupting the Smad signaling pathway and reducing CF fibrosis.

Interestingly, previous studies have shown that upregulation of HSPB5 expression in idiopathic pulmonary fibrosis (IPF) can reduce the expression of TRIM33 and weaken the interaction between TRIM33 and Smad4, thus hindering the ubiquitination and nuclear output of Smad4 [12]. As a member of the HSPB family and an important molecular chaperone, HSPB5 is involved in cytoskeleton stability, growth and differentiation, proliferation and cell migration and is closely related to the occurrence and development of a variety of diseases [30,31]. Previous studies have confirmed that inhibiting the interaction between TRIM33 and HSPB5 can restore the effect of TRIM33 on the TGF-β1 pathway and may be important for the treatment of IPF [12]. Similarly, we found that overexpression of HSPB5 in CFs decreased the expression of TRIM33 and further promoted Ang II–induced proliferation, migration and collagen synthesis, which could be reversed by overexpression of TRIM33.

## Conculsion

In the present study, we clarified that TRIM33 attenuated Ang II–induced myocardial fibrosis in vivo and in vitro by mediating TGF-β1/Smad3/4 signaling and that the effect of TRIM33 was regulated by HSPB5. Our studies delineate upstream and downstream mechanisms of TRIM33-mediated cardiac fibrosis and provide new insights into TRIM33-related treatment in cardiac fibrosis.
